# The Law of Attrition Revisited – Author’s Reply

**DOI:** 10.2196/jmir.8.3.e21

**Published:** 2006-09-29

**Authors:** Gunther Eysenbach

**Affiliations:** ^1^Centre for Global eHealth InnovationUniversity Health NetworkToronto ONCanada

## Author’s Reply

I agree with many of the points made by Christensen and Mackinnon in response to my “Law of Attrition” [1], which highlights the central role of adherence and exposure as important, but often underreported measures of eHealth interventions.

Recent articles in this journal, for example a paper by Danaher and colleagues on exposure measures in Web-based health behavior change programs [2], have picked up this discussion, and we are looking forward to receive more research explicating issues around sustained uptake of such interventions.

I do however not agree that focusing on attrition means focusing on the “negative” side of eHealth interventions. To formulate a “law of attrition” was partly motivated by the observation that many authors (the letter writer not included) are not very explicit about high dropout or nonusage rates in their study. Sometimes we have the impression that authors attempt to “hide” high attrition rates, perhaps fearing that reviewers and editors would deem a manuscript unpublishable if too many participants did not use an intervention or drop out from a trial. To explicate a “Law of Attrition” is an attempt to elucidate the fact that high dropout rates and nonusage seem common experiences for eHealth researchers and practitioners, and to encourage them to be forthcoming with such information, enabling them to cite a “law”. Attrition data should not be hidden or buried somewhere in the manuscript, but explicitly stated (already in the abstract) and - even better - analyzed using multivariate models. Participant characteristics, intervention attributes, as well as external variables need to be incorporated in such models, to analyze and predict events such as dropouts or nonusage. We will not be learning about what works and what does not by concealing such data.

Attrition measures are particularly important for the interpretation of “negative” studies (studies which do not show an effect on outcomes), as can be illustrated by a recently published study on electronic links between an emergency room and primary care physicians, which did not result in a significant reduction in resource utilization [3]. That study is a perfect example for the current tendency to focus on reporting traditional outcome measures (in this case, resource utilization in the emergency department and family physician offices), while failing to report any detailed exposure, adoption, or usage data. Without proper reporting and analysis of such data we will – in an “intention-to-use” analysis - never know whether it is the intervention which is principally flawed, or whether it was simply not (or not to a sufficient degree) adopted by the user group [4]. Adoption and sustained use are obvious prerequisites for any information and communication technology to change outcomes, and little is gained by just reporting “negative” outcomes without exploring why and for whom the technology worked (or failed to work) in terms of engagement, adherence, and continued use.
